# Colon Mucosa Exhibits Loss of Ectopic MUC5AC Expression in Patients with Ulcerative Colitis Treated with Oral Tacrolimus

**DOI:** 10.1155/2013/304894

**Published:** 2013-04-09

**Authors:** Tsutomu Mizoshita, Satoshi Tanida, Hironobu Tsukamoto, Keiji Ozeki, Takahito Katano, Masahide Ebi, Yoshinori Mori, Hiromi Kataoka, Takeshi Kamiya, Takashi Joh

**Affiliations:** Department of Gastroenterology and Metabolism, Nagoya City University Graduate School of Medical Sciences, 1-Kawasumi, Mizuho-cho, Mizuho-ku, Nagoya 467-8601, Japan

## Abstract

*Background*. Tacrolimus (FK506) is effective for patients with ulcerative colitis (UC). However, there are few reports on tacrolimus therapy (TT) with respect to the relationship with endoscopic and clinicopathologic findings. *Methods*. Thirty patients with moderate/severe active UC refractory to or dependent on corticosteroid were treated with oral tacrolimus. The expression of ectopic MUC5AC in the colon was pathologically analyzed before and at 12 weeks after TT, evaluating the Mayo score and steroid-sparing effects. *Results*. Both mean disease and endoscopic activity index scores were reduced at levels of statistical significance in 26 UC patients receiving more than one month of TT (*P* < 0.0001). The dose of prednisolone was reduced by a statistically significant amount (*P* = 0.00022), and 14 of the 26 patients (53.8%) had steroid-free status 12 weeks after TT. The decrease in ectopic MUC5AC expression in the mucous cells of the colon was significantly associated with endoscopic improvement of inflammation in the UC patients with TT (*P* = 0.043). Loss of ectopic MUC5AC expression was detected in all patients who had complete response. *Conclusions*. Tacrolimus appears to be effective for the treatment of moderate/severe UC patients. Loss of ectopic MUC5AC expression may be important for pathologic remission in the colon of UC patients.

## 1. Introduction

Ulcerative colitis (UC) is an idiopathic, chronic, and inflammatory disorder characterized by diarrhea, rectal bleeding, abdominal pain, fever, anemia, and body weight loss [1, 2]. Corticosteroid (CS) therapy is administered to patients with UC when flare-ups occur [1, 3]. Most patients with UC initially respond to CS therapy, but about 20% of patients become steroid dependent within 1 year after CS therapy starts [4]. Steroid-free status is important for patients with UC because CSs often induce undesirable side effects such as diabetes mellitus, osteoporosis, and opportunistic infections [2, 5]. 

Tacrolimus (FK506) is effective for patients with UC refractory to or dependent on CS and is usually used as a rescue and bridging therapy before initiating azathioprine (AZA) or 6-mercaptopurine (6-MP) therapy [6–12]. Tacrolimus therapy is useful as an alternative therapy for steroid-refractory UC [13]. Tacrolimus induces inhibition of the transcription of the early activation genes encoding interleukin- (IL-) 2, tumor necrosis factor-*α* (TNF-*α*), and interferon-*γ* (IFN-*γ*) that are responsible for the development of inflammation [6, 14]. A higher initial dose of tacrolimus has been observed to ensure achievement of target levels [15]. In a double-blind, placebo-controlled trial, Ogata et al. [13] showed that oral tacrolimus therapy in patients with steroid-refractory UC shortened the acute phase and induced rapid mucosal healing. However, there are few reports on tacrolimus therapy with respect to the relationship with endoscopic and clinicopathologic findings. We have previously shown the importance of gastric phenotypic expression for cancers of the digestive tract [16–18]. Several reports also have demonstrated ectopic gastric phenotypic expression, such as of MUC5AC, in the inflammatory bowel diseases [19, 20] and in UC-associated dysplasia/neoplasms [21, 22]. 

In the present study, we, therefore, analyzed the expression of ectopic MUC5AC in the mucous cells of the colon in patients with UC resistant to or untreatable with conventional therapy before and at 12 weeks after the start of oral tacrolimus therapy, using the Mayo score as a measure of disease activity and steroid-sparing effects of therapy.

## 2. Patients and Methods

### 2.1. Patients

Between August 2009 and June 2012, 30 patients with UC resistant to or untreatable with conventional therapy were administered oral tacrolimus at Nagoya City University Hospital, after informed consent was obtained. Of 30 patients with UC, 26 of them had received more than one month of tacrolimus therapy, while the tacrolimus therapy was stopped after less than 2 weeks in 4 cases. Before the start of treatment, infectious colitis, such as that caused by bacteria and cytomegalovirus, was ruled out by stool cultures, *Clostridium difficile* toxin testing, and pathological analysis of lesions. The extent of colonic involvement was determined by total colonoscopy [6]. Patients were classified as steroid resistant or steroid dependent, in accordance with the earlier published definitions [2, 6].

### 2.2. Symptoms and Endoscopic Assessment

Disease activity before and after oral tacrolimus therapy was measured using the Mayo score (also known as the disease activity index (DAI)) and the endoscopic activity index (EAI) [6, 23, 24]. Endoscopy was conducted within one week before oral tacrolimus administration, and a second endoscopic observation was performed for the evaluation of mucosal healing at 12 weeks after the patient was started on oral tacrolimus. As in a previous report [6], a complete response was defined as complete resolution of all symptoms (all assessment scores were zero). A partial response was defined as a reduction in the DAI of more than 4 points, with improvement in all categories, but not a complete response. In the event any assessment score was noted to worsen or remain unchanged despite improvement in other scores, the patient was considered to have treatment failure. Patients whose symptoms worsened at any time or did not improve after more than one week (in the case of a total DAI score ≥10 at baseline) were considered to have had treatment failure if the investigators assessed that the tacrolimus therapy could not be continued [6]. The DAI was evaluated at weeks 0 and 12 after administration of oral tacrolimus. 

### 2.3. Treatment

Tacrolimus was administered in its oral formulation [2, 10, 14]. According to the Japanese protocol, the dosage was adjusted to produce trough tacrolimus whole-blood levels of 10 to 15 ng/mL to induce remission. After inducing clinical remission, tacrolimus whole-blood trough concentrations were maintained at a lower level, between 5 and 10 ng/mL [2, 10, 14]. Tacrolimus is not currently approved in Japan for maintenance therapy; therefore, tacrolimus administration was stopped three months after the patient was started on oral tacrolimus [14]. 

### 2.4. Immunohistochemistry

In 18 UC patients of 26 cases who had received more than one month of tacrolimus therapy, biopsies from the inflamed mucosa in the colon were obtained before and at 12 weeks after administration of tacrolimus to evaluate the histology and the MUC5AC expression when endoscopy was performed. MUC5AC expression is generally detected in the cytoplasm of mucous cells of the stomach, while no MUC5AC expression is observed in the normal colon. Immunohistochemical staining in the biopsy samples from the colons of the UC patients receiving tacrolimus medication was carried out with the following monoclonal antibody: MUC5AC (CLH2; 1:500, Novocastra Laboratories, Newcastle, UK). The precise procedures for immunohistochemical techniques were as described previously [16, 17]. Briefly, 4-*μ*m-thick consecutive sections were deparaffinized and hydrated through a graded series of ethanols. After inhibition of endogenous peroxidase activity by immersion in 3% H_2_O_2_ methanol solution, sections were incubated with the primary antibody, washed thoroughly in phosphate-buffered saline (PBS), and then incubated with biotinylated secondary antibody followed by the avidin-biotinylated horseradish peroxidase complex (Vectastain Elite ABC kit; Vector Laboratories, Burlingame, CA, USA). Finally, immune complexes were visualized by incubation with 0.01% H_2_O_2_ and 0.05% 3,3′-diaminobenzidine tetrachloride (DAB). Nuclear counterstaining was accomplished with Mayer's hematoxylin.

Two independent investigators (TM and HT) judged the histology and immunohistochemical staining of MUC5AC in the cytoplasm of mucous cells of the colon, as previously described [18].

### 2.5. Statistical Analyses

Regarding statistical analyses before and after tacrolimus administration, the Wilcoxon *t*-test was applied to establish the significance of differences in the DAI and EAI scores and the steroid-sparing effects. The relationship between MUC5AC expression and the EAI was assessed using Fischer's exact test. *P*  values < 0.05 were considered to be statistically significant.

## 3. Results

### 3.1. Patient Characteristics

Of 30 patients with UC, 26 patients received tacrolimus therapy for more than one month, while the tacrolimus therapy was stopped after less than 2 weeks in 4 patients. Adverse events caused tacrolimus withdrawal in 3 patients, while 1 patient with UC exhibited the fulminant type, experiencing uncontrolled continuous bleeding from the inflammatory colon mucosa, which resulted in colectomy in less than two weeks after the start of tacrolimus therapy.

The baseline characteristics of the 26 patients receiving more than one month of tacrolimus therapy are shown in Table 1. The male/female ratio was 12/14, and the median ages at diagnosis and the start of therapy were 37.4 years (16–78 years) and 43.6 years (17–81 years) (median (range)), respectively. The median disease duration was 6.1 years (0.5–21 years) (median (range)). The 26 cases were divided into 16 extensive and 10 left-sided types of UC, and they were also classified as 3 steroid-resistant and 23 steroid-dependent types of UC. Regarding concomitant medication, 19 patients received prednisolone (≥10 mg/day), 23 received 5-aminosalicylates, 4 received immunosuppressants (AZA or 6-MP), 1 received infliximab, and 23 received granulocyte and monocyte adsorptive (GMA) therapies (Table 1). 

### 3.2. DAI and EAI Scores

**Figure 1:**
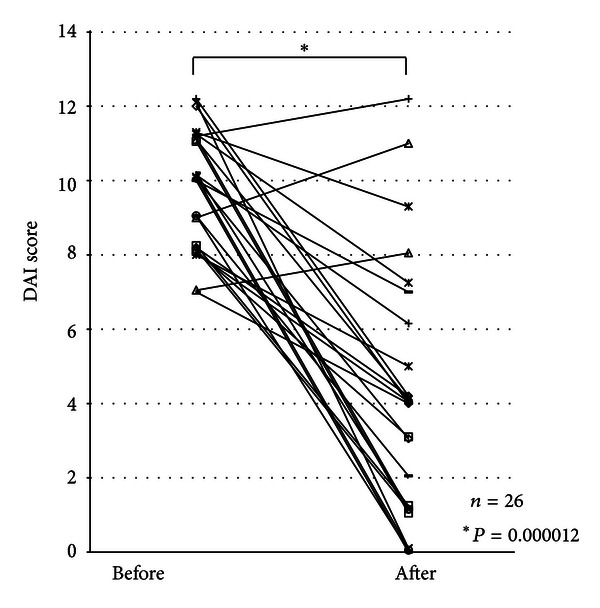
Disease activity index (DAI) scores before and at 12 weeks after oral tacrolimus therapy.

The mean DAI score was significantly reduced from 9.65 ± 0.30 (average ± SE) at the start of oral tacrolimus therapy to 3.92 ± 0.67 at week 12 in 26 patients receiving more than one month of tacrolimus therapy (*P* = 0.000012, Figure 1). Four patients had a complete response, and 14 patients had a partial response to the tacrolimus therapy. However, the remaining 8 patients had treatment failure. Of the 8 patients with treatment failure, 6 received additional infliximab therapy, while 2 patients were given an increased dosage of prednisolone.

**Figure 2:**
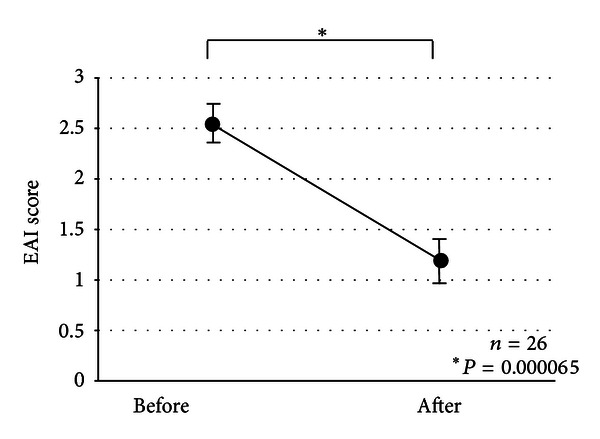
Endoscopic activity index (EAI) scores before and at 12 weeks after oral tacrolimus therapy.

The mean EAI score was significantly reduced from 2.53 ± 0.11 (average ± SE) at the start of oral tacrolimus medication to 1.19 ± 0.18 at week 12 in 26 patients receiving more than one month of tacrolimus therapy (*P* = 0.000065, Figure 2). Seven patients experienced endoscopic complete remission (mucosal healing), and 12 patients had a decrease in their EAI scores at 12 weeks after the start of tacrolimus therapy, compared with their EAI scores before receiving tacrolimus. However, improvement in the inflammatory mucosa in the colon was not observed endoscopically in the remaining 7 patients.

### 3.3. Steroid-Sparing Effects of Tacrolimus Therapy

**Figure 3:**
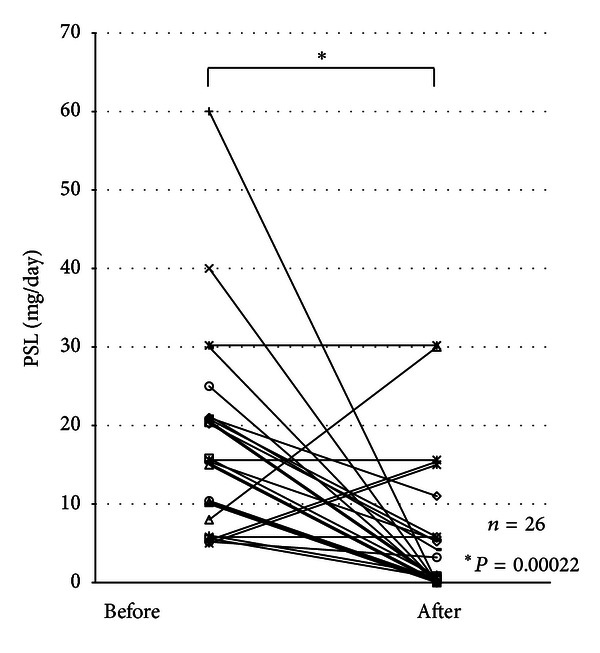
Dosage of prednisolone (mg/day) before and at 12 weeks after oral tacrolimus therapy.

The mean dose of prednisolone was significantly reduced from 17.2 ± 2.5 (mg/day, average ± SE) at the start of oral tacrolimus medication to 5.4 ± 1.7 at week 12 in 26 patients receiving more than one month of tacrolimus therapy (*P* = 0.00022, Figure 3). Fourteen of 26 patients (53.8%) had steroid-free status at 12 weeks after the start of oral tacrolimus. Of the remaining 12 patients, 6 patients had their dose of oral prednisolone decreased at 12 weeks after the start of tacrolimus therapy, compared with the dose before therapy. The other 6 patients had no change or increase in their dose of oral prednisolone at 12 weeks after the start of tacrolimus therapy, compared with the dose before therapy.

### 3.4. Relationships between MUC5AC Expression, EAI, and DAI

**Figure 4:**
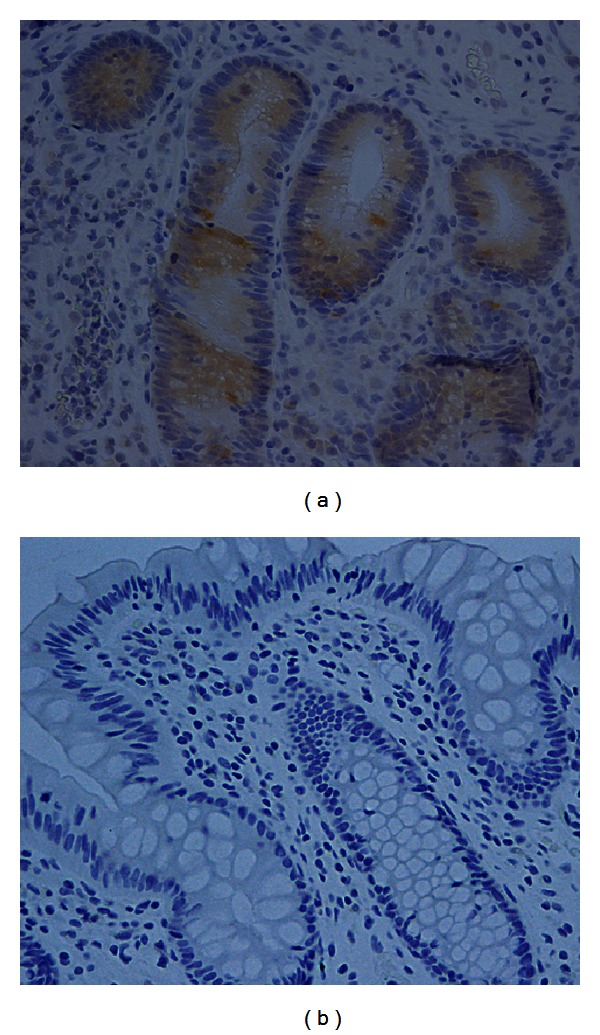
Ectopic MUC5AC expression is often positive in the cytoplasm of the mucous cells of the colon in during active UC (a). Loss of MUC5AC expression is produced by tacrolimus in patients with complete response (b). (a) and (b): ×400.

The relationships between MUC5AC expression, EAI, and DAI are summarized in Table 2. The decrease in ectopic MUC5AC expression in the mucous cells of the colon was significantly associated with endoscopic improvement of inflammation in the UC patients receiving tacrolimus (*P* = 0.043, Table 2). In 3 UC patients with complete response, all of them showed marked improvement of the mucosal inflammation in the colon, suggesting the relation between loss of MUC5AC expression and endoscopic remission as evidenced by mucosal healing (Table 2, Figure 4). In 9 patients with partial response, 6 (66.7%) of the 9 patients had cytoplasmic MUC5AC expression in the mucous cells of the colon before tacrolimus administration, and a decrease in MUC5AC expression was produced by the tacrolimus in all 6 of these patients (Table 2). In the remaining 3 patients with partial response, no MUC5AC expression was detected in the mucous cells of the colon before tacrolimus administration, and in 2 of those patients, no immunohistochemical staining of MUC5AC was retained at 12 weeks after receiving tacrolimus. However, the remaining single patient had an increase in MUC5AC expression score after tacrolimus administration. In the 6 patients with treatment failure, increase or no decrease of MUC5AC cytoplasmic expression in the mucous cells of the colon was observed in 5 (83.3%) of the 6 patients, in spite of receiving tacrolimus. The remaining single patient had a decrease in MUC5AC expression; however, no improvement in the EAI score was observed endoscopically.

### 3.5. Adverse Events

Table 3 shows the frequency of adverse events during tacrolimus therapy. Seven (23.3%) of the 30 study subjects experienced at least one adverse event. Tacrolimus withdrawal was necessary in 3 (10.0%) of the 30 subjects. Among the 3 patients requiring tacrolimus withdrawal, one patient had both liver function disorder and nausea, another patient had liver function disorder, and the third patient had nausea, resulting in less than 2 weeks of tacrolimus administration. Regarding the remaining 4 patients who experienced adverse events, 3 patients developed tremor, and 1 patient had both tremor and chest pain, being unnecessary to stop the tacrolimus therapy.

## 4. Discussion

The results of the present study give clear evidence that loss/reduction of ectopic MUC5AC cytoplasmic expression in the mucous cells of the colon is strongly associated with endoscopic remission in patients with UC. Loss of ectopic MUC5AC expression was detected in all study subjects with complete response (Table 2, Figure 4). The gel-forming mucins (particularly MUC5AC and MUC6) may have a role in epithelial wound healing after mucosal injury in inflammatory bowel disease, in addition to providing mucosal protection [19]. The patients with UC had levels above the threshold, and their mucosae were strongly labeled with anti-M1/MUC5AC antibody by immunohistochemistry [25]. MUC5AC and TFF1 expression in goblet cells is common in inflammatory bowel disease and other inflammatory conditions of the colon, suggesting that these changes may represent a nonspecific repair function of the colon cells to compensate for damage to barrier function [20]. The presence of MUC5AC correlated positively with inflammatory activity in UC [26]. Expression of gastric differentiation markers is potentially useful for the detection of UC-associated dysplasia, suggesting that the expression of gastric phenotype in the colon is important for UC-associated colorectal carcinogenesis [22]. Gastric-type mucins may be useful in the differential diagnosis between UC-associated neoplasms and sporadic neoplasms [21]. Watanabe et al. [27] found that patients with UC-associated colorectal cancers had poorer survival than patients with sporadic cancers in the advanced stages in the Japanese population. In the aggressive type of sporadic colorectal carcinomas, several reports have shown the importance of MUC5AC expression [18, 28]. Taking into account the above-mentioned previous reports and our present data, we consider that loss of ectopic MUC5AC expression may be important in achieving pathologic remission status in the colon of UC patients. However, there is the possibility that MUC5AC is decreased because remission was achieved. Further studies may be needed to clarify whether reduction of ectopic MUC5AC expression affects positively remission status or not.

Our study results have provided clear evidence that tacrolimus induces a remarkable decrease in DAI and EAI scores in patients with UC. Eighteen (69.2%) of 26 patients had improvement in their DAI scores, and 7 patients had mucosal healing, as shown by endoscopy. In a double-blind, placebo-controlled trial, Ogata et al. [6] found a clinical response rate at week 2 of 52.5% in the tacrolimus groups (high and low trough groups) and 10.0% in the placebo group. In the high trough group, improvement in DAI scores occurred in 68.4% of cases [6]. Tacrolimus may be effective for short-term clinical improvement in patients with refractory UC [29]. Maintenance therapy with tacrolimus for patients with UC could be considered an alternative to thiopurine therapy [2]. Long-term administration of tacrolimus appears to be an effective, well-tolerated, and safe treatment for patients with refractory UC [7, 10]. We consider that tacrolimus has a strong remission-induction effect in the moderate/severe stages of UC. 

We also have shown that the steroid-sparing effects of tacrolimus enable a remarkable reduction in the dose of prednisolone in UC patients. Fourteen of 26 patients (53.8%) obtained steroid-free status at 12 weeks after the start of oral tacrolimus administration. Tacrolimus offers benefits to selected patients with steroid-dependent UC, including patients who are intolerant of AZA or 6-MP [12]. Oral tacrolimus is effective in patients with steroid-refractory UC [11, 13, 30, 31]. Studies have shown that CS therapy could be discontinued or tapered in most UC patients treated with a CS at the initiation of tacrolimus therapy, suggesting its long-term effect [7, 10]. We consider that tacrolimus induces steroid-free remission or enables a remarkable reduction in steroid dosage in the moderate/severe stages of UC.

Infliximab offers a therapeutic option as rescue therapy in about one-fourth of patients with active UC who do not respond to tacrolimus [32]. Infliximab can induce remission in patients with UC who do not tolerate or respond to tacrolimus therapy [33]. Among our study subjects, 8 patients had treatment failure in spite of tacrolimus therapy. Of the 8 patients with treatment failure, 6 of them received the additional infliximab therapy, while 2 of the patients were given increased doses of prednisolone. 

Regarding adverse events of tacrolimus, finger tremor is the most common adverse event. Other adverse events, such as renal function impairment, infection, headache, nausea, and arthralgia, are sometimes experienced [10, 34]. Among our study subjects, 7 (23.3%) of 30 patients experienced at least one adverse event. Tacrolimus withdrawal was necessary in 3 (10.0%) of the 30 subjects, while in the remaining 4 patients, it was not necessary to stop the tacrolimus therapy. 

Among the UC patients in the present study who received tacrolimus therapy, 23 (88.5%) of 26 patients received GMA (Adacolumn; JIMRO Co., Ltd., Takasaki, Gunma, Japan) therapy preceding the tacrolimus therapy. As a calcineurin inhibitor, tacrolimus induces inhibition of the transcription of the early activation genes encoding IL-2, TNF-*α*, and IFN-*γ* that are responsible for the development of inflammation [6, 14]. On the other hand, GMA is an efficacious therapeutic option for refractory inflammatory bowel disease [35–38]. GMA has few side effects [38], suggesting that there are the different action points between tacrolimus and GMA. GMA depletes elevated and activated myeloid lineage leukocytes and has been associated with marked down-regulation of inflammatory cytokines including IL-1*β*, IL-6, IL-8, and TNF-*α*, which are released by myeloid leukocytes and lymphocytes, most likely via an upstream mechanism that involves adsorption of cytokine-producing cells [37, 39–41]. Intensive GMA treatment at a rate of two GMA sessions per week has recently been shown to be more effective than conventional weekly GMA in patients with refractory UC [42]. Moreover, intensive GMA treatment safely induces rapid remission in patients with moderately active UC inflammation [42], and GMA therapy at an early stage significantly reduces steroid administration and steroid-dependency in the long-term clinical course in patients with their first UC episode [43]. Regarding induction therapy for remission in the active stage of UC, there may be synergistic curative effects of tacrolimus and GMA, since these therapies have different action points. 

In conclusion, tacrolimus appears to be an effective treatment for patients with UC who are resistant to or cannot be treated with conventional therapy. In addition, loss of ectopic MUC5AC expression may be important for the goal of pathologic remission status in the colon of patients with UC.

## Figures and Tables

**Table 1:** Patients' baseline characteristics (*n* = 26).

Sex (male/female)	12/14
Age at diagnosis (median (range)) (years)	37.4 (16–78)
Age at start of the therapy (median (range)) (years)	43.6 (17–81)
Disease duration (median (range)) (years)	6.1 (0.5–21)
Extent of disease	
Extensive (%)	16 (61.5%)
Left sided (%)	10 (38.5%)
Response to corticosteroids	
Steroid resistance (%)	3 (11.5%)
Steroid dependent (%)	23 (88.5%)
Concomitant medication	
Predonisolone (≥10 mg/day)	19
5-Aminosalicylates	23
Immunosuppressants (AZA or 6-MP)	4
Infliximab	1
GMA	23

AZA: azathioprine; 6-MP: 6-mercaptopurine; GMA: granulocyte and monocyte adsorptive apheresis.

**Table 2:** The relations between MUC5AC immunostaining, EAI, and DAI in the UC patients with tacrolimus treatment (*n* = 18).

	MUC5AC*			EAI			Response
	(Scores)			(Scores)			
	Before		After	Before		After
Case 1	1	→	0	3	→	0	Complete response
Case 2	1	→	0	3	→	0	Complete response
Case 3	1	→	0	2	→	0	Complete response

Case 4	2	→	1	3	→	0	Partial response
Case 5	2	→	1	3	→	0	Partial response
Case 6	2	→	1	2	→	0	Partial response
Case 7	1	→	0	3	→	1	Partial response
Case 8	1	→	0	2	→	1	Partial response
Case 9	1	→	0	2	→	1	Partial response
Case 10	0	→	0	3	→	1	Partial response
Case 11	0	→	0	2	→	1	Partial response
Case 12	0	→	2	3	→	2	Partial response

Case 13	3	→	3	2	→	3	Treatment failure
Case 14	2	→	2	2	→	2	Treatment failure
Case 15	1	→	2	3	→	3	Treatment failure
Case 16	1	→	1	2	→	2	Treatment failure
Case 17	1	→	0	2	→	2	Treatment failure
Case 18	0	→	3	1	→	2	Treatment failure

EAI: endoscopic activity index; DAI: disease activity index. *MUC5AC versus EAI, *P* = 0.043 with Fischer exact probability.

**Table 3:** Adverse events that developed during tacrolimus treatment (*n* = 30).

Adverse events	Cases (%)
Tremor	4 (13.3%)
Liver function disorder	2 (6.7%)
Nausea	2 (6.7%)
Chest pain	1 (3.3%)
